# Prognostic impact of polypharmacy by drug essentiality in patients on hemodialysis

**DOI:** 10.1038/s41598-021-03772-0

**Published:** 2021-12-20

**Authors:** Mineaki Kitamura, Kosei Yamaguchi, Yuki Ota, Satoko Notomi, Maya Komine, Rika Etoh, Takashi Harada, Satoshi Funakoshi, Hiroshi Mukae, Tomoya Nishino

**Affiliations:** 1 Department of Nephrology, Nagasaki University Graduate School of Biomedical Sciences, Nagasaki, Japan; 2 Nagasaki Renal Center, Nagasaki, Japan; 3 Department of Nephrology, Sasebo City General Hospital, Nagasaki, Japan; 4 Department of Respiratory Medicine, Nagasaki University Graduate School of Biomedical Sciences, Nagasaki, Japan

**Keywords:** Risk factors, Renal replacement therapy, Outcomes research, Pharmacology, Drug safety

## Abstract

Although polypharmacy is common among patients on hemodialysis (HD), its association with prognosis remains unclear. This study aimed to elucidate the association between the number of prescribed medicines and all-cause mortality in patients on HD, accounting for essential medicines (i.e., antihypertensives, antidiabetic medicines, and statins) and non-essential medicines. We evaluated 339 patients who underwent maintenance HD at Nagasaki Renal Center between July 2011 and June 2012 and followed up until June 2021. After adjusting for patient characteristics, the number of regularly prescribed medicines (10.0 ± 4.0) was not correlated with prognosis (hazard ratio [HR]: 1.01, 95% confidence interval [CI] 0.97–1.05, p = 0.60). However, the number of non-essential medicines (7.9 ± 3.6) was correlated with prognosis (HR: 1.06, 95% CI 1.01–1.10, p = 0.009). Adjusting for patient characteristics, patients who were prescribed more than 10 non-essential medicines were found to have a significantly higher probability of mortality than those prescribed less than five non-essential medicines, with a relative risk of 2.01 (p = 0.004). In conclusion, polypharmacy of non-essential medicines increases the risk of all-cause mortality in patients on HD. As such, prescribing essential medicines should be prioritized, and the clinical relevance of each medicine should be reviewed by physicians and pharmacists.

## Introduction

Polypharmacy is common among patients with chronic kidney disease (CKD), particularly those undergoing hemodialysis (HD)^1–6^, because of the presence of various comorbid conditions, such as hypertension and cardiovascular diseases^1^. Moreover, more than half of patients undergoing HD develop end-stage renal disease secondary to diabetes mellitus^7^. Therefore, patients on HD are prescribed various medicines to treat multiple comorbidities, and compared to any other medical subspecialty, patients treated by nephrologists have a more complex clinical presentation^6^. Polypharmacy is defined as prescribing several medicines to patients; this is a critical practice as it can cause drug-drug interactions and harm patients^8^. Although the number of medicines for defining polypharmacy varies^8^, patients on HD are prescribed approximately 10 medicines^3,4^, and this situation was defined as excessive polypharmacy in previous studies^3,8^.

A systematic review showed that the number of prescribed medicines is correlated with patient prognosis in the general population^9^. However, only a few studies have investigated the association between the number of prescribed medicines and mortality in patients on HD^10^. A previous study on 152 patients who were followed up for approximately 3 years found no significant association between polypharmacy and all-cause mortality in the HD population^10^. However, this was probably due to the short-term follow-up, small sample size, and many confounding factors that hindered the elucidation of the actual prognostic impact of polypharmacy in patients receiving HD. Thus, the interpretation of the polypharmacy impact on patients undergoing HD requires further analyses.

Some medicines for comorbidities in patients on HD have been shown to improve prognosis. Antihypertensive medicines can prevent cardiovascular diseases and have prognostic benefits in patients undergoing HD^11^. For example, amlodipine has been shown to prevent cardiovascular diseases in these patients^12^. Antidiabetic medicines also play an important role as glycemic control is crucial in diabetes patients on HD^13^. Dipeptidyl peptidase-4 inhibitors allow good glycemic control^14^. A 5-year observational study showed that antidiabetic medicines reduce the risk of death in diabetes patients on maintenance HD^15^. Further, atorvastatin reportedly exerts a favorable prognostic effect in patients on HD^16^. Our previous study also found a positive effect of pitavastatin on mortality in patients undergoing HD^17^.

However, the association between polypharmacy and prognosis in the context of essential medicines (e.g., antihypertensive and antidiabetic medicines) and non-essential medicines in patients on HD remains unclear. We hypothesized that the number of prescribed non-essential medicines is correlated with prognosis in these patients. Thus, this study aimed to elucidate the association between the number of prescribed medicines and all-cause mortality in patients receiving HD, considering the essential medicines.

## Results

### Patient characteristics and trends in medicine prescription

**Figure 1:**
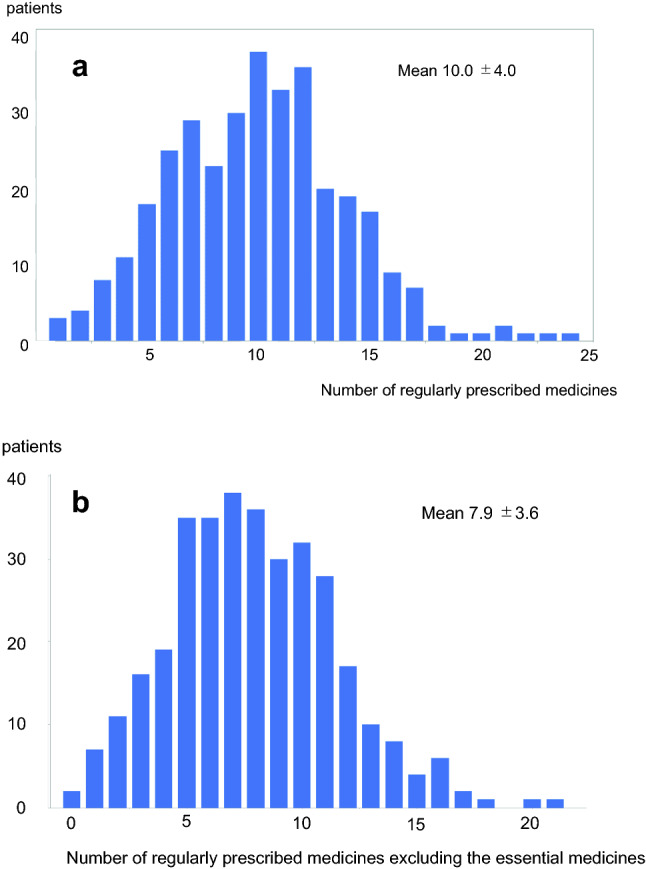
Histogram showing the number of medicines regularly prescribed to patients on hemodialysis. (**a**) Overall number of medicines. (**b**) Non-essential medicines. The patients were prescribed 10 medicines on average, and approximately 20% of these medicines were categorized as essential medicines. Statistical analyses were performed using the JMP Pro 15.0.0 (3903308).

A total of 339 patients were evaluated; among them, 57% were men. The mean age was 67.3 ± 13.3 years, and the median duration of dialysis was 4.7 (interquartile range [IQR] 1.9–10.1) years. The mean hemoglobin A1c level, which was only available in patients with diabetes, was 5.8 ± 1.2%. The mean numbers of prescribed essential and non-essential medicines were 10.0 ± 4.0 and 7.9 ± 3.6, respectively (Fig. 1a-b).

In total, 151 and 188 patients were divided into two groups: those prescribed < 10 medicines and those prescribed ≥ 10 medicines. The patient characteristics according to group are listed in Table 1. Patients were also categorized into two groups: those prescribed < 8 non-essential medicines and those prescribed 8 ≥ non-essential medicines; their characteristics are listed in Supplementary Table 1. The prescription trend is shown in Supplementary Table 2. The proportions and categories of prescribed medicines in all patients are shown in Supplementary Table 3.

**Table 1:** Patient characteristics by total number of prescribed medicines.

	Less than 10 (N = 151)	At least 10 (N = 188)	p-value
Age (years)	67.1 ± 14.5	67.6 ± 12.3	0.73
Male (%)	58.3	56.4	0.73
Dialysis vintage^a^ (years)	4.0 (1.3–9.5)	5.0 (2.3–10.5)	0.048
Dialysis time^a^ (h)	4 (3–4)	4 (3–4)	0.45
Hypertension (%)	82.8	86.1	0.40
Diabetes mellitus (%)	25.2	42.0	0.001
Ischemic heart disease (%)	24.5	42.0	< 0.001
Valve replacement therapy (%)	2.7	4.3	0.42
Cerebral hemorrhage (%)	8.0	5.3	0.33
Cerebral infarction (%)	23.8	25.5	0.72
Arteriosclerosis obliterans (%)	13.3	19.7	0.11
Cardiothoracic ratio (%)	51.6 ± 6.2	52.7 ± 5.4	0.09
Dry weight (kg)	51.3 ± 10.4	52.6 ± 11.5	0.29
Systolic blood pressure (mmHg)	147 ± 23	152 ± 25	0.06
Left ventricular ejection fraction (%)	65 ± 10	65 ± 10	0.69
Hemoglobin (g/dL)	10.7 ± 1.3	10.9 ± 1.4	0.33
Ferritin^a^ (ng/mL)	66.3 (25.6–180.0)	63.3 (23.7–199.7)	0.97
Transferrin saturation (%)	26.1 ± 15.4	24.0 ± 12.6	0.17
Albumin (g/dL)	3.5 ± 0.4	3.6 ± 0.4	0.02
Corrected calcium (mg/dL)	9.3 ± 0.8	9.2 ± 0.8	0.52
Phosphate (mg/dL)	5.4 ± 1.5	5.8 ± 1.7	0.04
Intact-parathyroid hormone^a^ (pg/mL)	77 (30–162)	69 (28–137)	0.29
Alkaline phosphatase^a^ (IU/L)	252 (192–336)	250 (191–341)	0.93
Blood urea nitrogen (mg/dL)	66.8 ± 18.9	69.1 ± 17.5	0.23
Creatinine (mg/dL)	9.8 ± 3.5	10.6 ± 3.3	0.04
Total cholesterol (mg/dL)	157 ± 37	164 ± 37	0.07
Triglycerides^a^ (mg/dL)	81 (61–124)	98 (69–136)	0.02
C-reactive protein^a^ (mg/dL)	0.21 (0.07–0.64)	0.16 (0.05–0.46)	0.28

Data are expressed as the mean ± standard deviation, ^a^ median (interquartile range).

The t-test or Mann–Whitney U test were used in the analysis.

Multivariable logistic regression analysis showed a tendency toward a higher number of prescriptions in patients with a history of ischemic heart disease (odds ratio [OR]: 1.98, 95% confidence interval [CI]: 1.22–3.22, p = 0.005) and those with diabetes mellitus (OR: 2.00, 95% CI: 1.24–3.25, p = 0.004). The results of the logistic regression model are presented in Supplementary Table 4.

### Survival analysis

**Figure 2:**
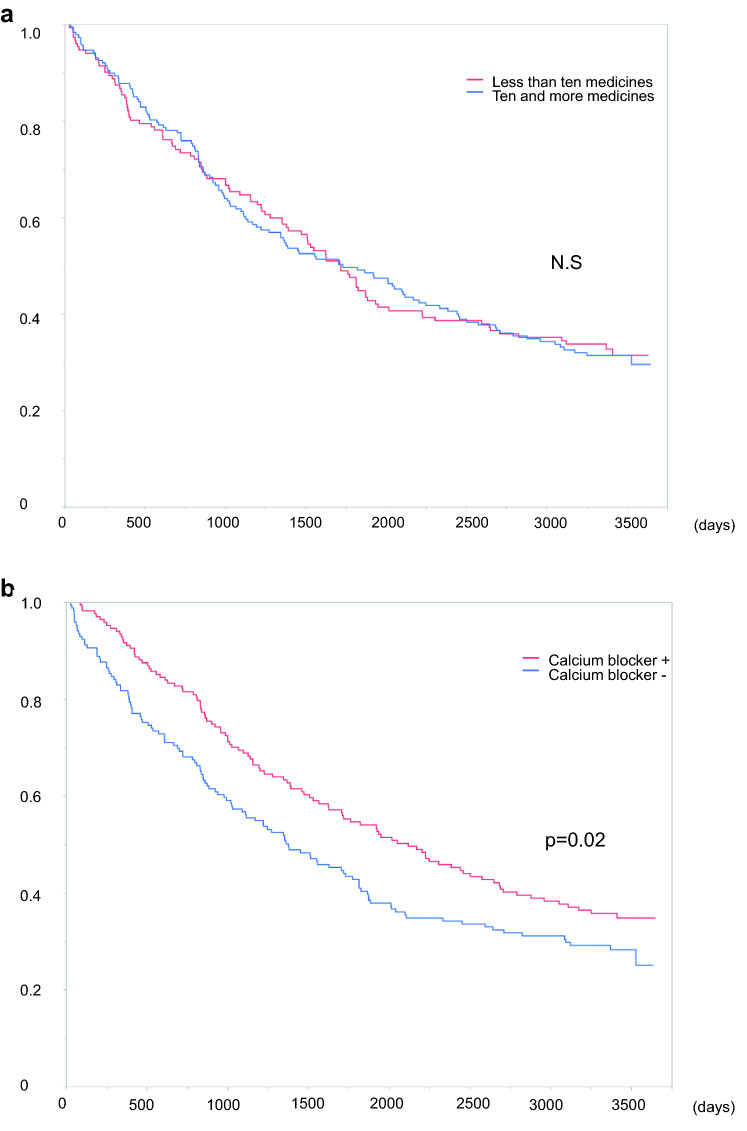
Kaplan–Meier survival curves. (**a**) Survival curve by the total number of prescribed medicines. There was no significant difference between the two groups (p = 0.67). (**b**) Survival curve by prescription of calcium channel blockers (yes, n = 169; no, n = 170). Patients who were prescribed calcium channel blockers had better prognoses than those not prescribed calcium channel blockers (p = 0.02). Statistical analyses were performed using the JMP Pro 15.0.0 (3903308).

Of 339 patients, 226 patients died during the follow-up period. The median follow-up period was 4.5 (IQR: 1.8–9.2) years and 4.1 (IQR: 2.0–9.1) years in the < 10 and ≥ 10 medicines groups, respectively. There was no significant difference in mortality between the < 10 and ≥ 10 medicines groups (64.2% vs. 66.5%, p = 0.67). The Kaplan–Meier curve also showed no significant difference in prognosis between the two groups (p = 0.92) (Fig. 2a). As an example of the effect of essential medicines on prognosis, patients prescribed calcium channel blockers (CCBs) had significantly better prognoses than those who were not prescribed CCBs (p = 0.03; Fig. 2b).

### Cox regression analyses

Univariable Cox regression analysis to elucidate the association between patient characteristics and prognosis showed that the total number of prescribed medicines (p = 0.56) and the number of prescribed non-essential medicines (p = 0.50) were not associated with prognosis (Supplementary Table 5). In the multivariable Cox analyses, both Model 1 (used to evaluate the total number of prescribed oral medicines) and Model 2 (included the number of prescribed non-essential medicines) showed that age, sex, dialysis time, diabetes mellitus, stroke history, cardiothoracic ratio, albumin level, serum phosphate level, and creatinine level were associated with prognosis. After adjusting for clinically important confounding factors, the number of the total prescribed medicines did not correlate with prognosis (hazard ratio [HR]: 1.01, 95% CI: 0.97–1.05, p = 0.60). However, the number of prescribed non-essential medicines correlated with prognosis (HR: 1.06, 95% CI: 1.01–1.10, p = 0.009) (Table 2). According to sensitivity analyses, the number of prescribed non-essential medicines was not associated with prognosis in patients with diabetes mellitus (HR: 0.99, 95% CI: 0.92–1.05, p = 0.66) (Supplementary Table 6). On the other hand, the number of prescribed non-essential medicines correlated with prognosis in patients with ischemic heart diseases (HR: 1.05, 95% CI: 1.01–1.17, p = 0.03) (Supplementary Table 7).

**Table 2:** Multivariable cox proportional regression models on the influencing factors of prognosis.

	Model 1			Model 2		
	HR	95% CI	p-value	HR	95% CI	p-value
Age/years	1.03	1.02–1.05	< 0.001	1.03	1.02–1.05	< 0.001
Male vs female	1.68	1.19–2.38	0.004	1.80	1.19–2.38	< 0.001
Dialysis vintage/year	1.01	0.99–1.03	0.34	1.01	0.99–1.03	0.50
Dialysis time/hour	0.61	0.44–0.85	0.003	0.59	0.43–0.82	0.002
Diabetes mellitus	1.51	1.08–2.09	0.01	1.42	1.08–2.09	0.03
IHD history	1.17	0.87–1.57	0.29	1.14	0.87–1.57	0.39
Stroke history	1.39	1.03–1.88	0.03	1.41	1.03–1.88	0.02
Cardiothoracic ratio/%	1.04	1.01–1.06	0.002	1.04	1.02–1.07	0.001
Dry weight/kg	0.99	0.97–1.01	0.44	0.99	0.97–1.01	0.42
Systolic BP/10 mmHg	0.95	0.90–1.01	0.09	0.96	0.90–1.01	0.15
Hemoglobin/g/dL	1.09	0.98–1.21	0.12	1.08	0.98–1.20	0.15
Albumin/g/dL	0.41	0.27–0.62	< 0.001	0.42	0.28–0.62	< 0.001
Corrected calcium/mg/dL	1.14	0.92–1.40	0.23	1.12	0.91–1.38	0.27
Phosphate/mg/dL	1.12	1.02–1.23	0.01	1.12	1.02–1.23	0.02
Intact PTH/10 pg/mL	1.00	0.98–1.01	0.62	1.00	0.98–1.01	0.59
BUN/10 mg/dL	1.02	0.93–1.12	0.65	1.04	0.94–1.14	0.47
Creatinine/mg/dL	0.91	0.86–0.97	0.003	0.90	0.85–0.96	0.001
Vitamin D	0.88	0.64–1.20	0.41	0.84	0.64–1.20	0.26
Phosphate binders	0.81	0.59–1.11	0.19	0.78	0.59–1.11	0.12
No. of total drugs	1.01	0.97–1.05	0.60			
No. of non-essential drugs				1.06	1.01–1.10	0.009

Model 1: Including the total number of prescribed drugs.

Model 2: Including the number of prescribed non-essential drugs.

IHD, ischemic heart disease; BP, blood pressure; intact PTH, intact-parathyroid hormone; BUN, blood urea nitrogen; No., number; non-essential drugs: all prescribed drugs excluding anti-hypertensive drugs, diuretics, anti-diabetes drugs, and statins.

### Effect of medicines on prognosis

Univariable Cox regression models to elucidate the effect of medicines on prognosis showed that CCBs and statins had a positive correlation, whereas oral vasopressors had a negative correlation with prognosis (Table 3). After adjusting for clinically important patient characteristics, angiotensin receptor blockers (ARBs) and other antihypertensive drugs, as well as anti-diabetes drugs, were associated with improved prognosis in addition to CCBs and statins. In contrast, histamine 2 blockers and other gastrointestinal medicines worsened prognosis in addition to oral vasopressors (Table 3).

**Table 3:** Univariable and multivariable-adjusted Cox proportional regression models for drugs influencing patient prognosis.

	Univariable			Multivariable		
	HR	95% CI	p-value	HR	95% CI	p-value
Angiotensin receptor blockers	0.79	0.60–1.03	0.07	0.58	0.44–0.78	0.01
Beta blockers	0.81	0.57–1.15	0.22	0.97	0.66–1.43	0.89
Calcium blockers	0.74	0.57–0.96	0.02	0.58	0.44–0.78	< 0.001
Diuretics	0.95	0.72–1.25	0.71	0.94	0.68–1.30	0.72
Other antihypertensive drugs	0.93	0.63–1.37	0.71	0.60	0.40–0.91	0.02
Antiplatelets	1.28	0.98–1.68	0.07	1.07	0.79–1.46	0.64
Antidiabetics	0.90	0.63–1.38	0.63	0.52	0.32–0.87	0.01
Proton pump inhibitors	1.08	0.83–1.41	0.57	0.74	0.55–1.00	0.053
H_2_ blockers	1.14	0.85–1.54	0.38	1.50	1.09–2.07	0.01
Other gastrointestinal medicines	0.91	0.70–1.18	0.48	1.38	1.00–1.91	0.048
Laxatives	1.13	0.87–1.47	0.36	0.76	0.57–1.03	0.08
Cinacalcet	0.39	0.25–0.61	< 0.001	0.83	0.49–1.41	0.49
Sleeping medicines	1.08	0.80–1.47	0.62	1.06	0.75–1.52	0.73
Anti-epileptic, parkinsonism, and depression medicines	0.88	0.62–1.27	0.50	0.79	0.48–1.10	0.13
Statins	0.55	0.37–0.83	< 0.001	0.50	0.32–0.77	0.002
Oral vasopressors	1.72	1.30–2.28	< 0.001	1.51	1.10–2.07	0.01

The adjusted values were calculated by adjusting for age, sex, dialysis vintage, dialysis time, ischemic heart disease history, stroke history, cardiothoracic ratio, dry weight, systolic blood pressure, hemoglobin, serum albumin, corrected calcium, intact-parathyroid hormone, blood urea nitrogen, serum creatinine, prescription of vitamin D, and prescription of phosphate binders.

### The prognosis for each quantile of the number of prescribed medicines

**Figure 3:**
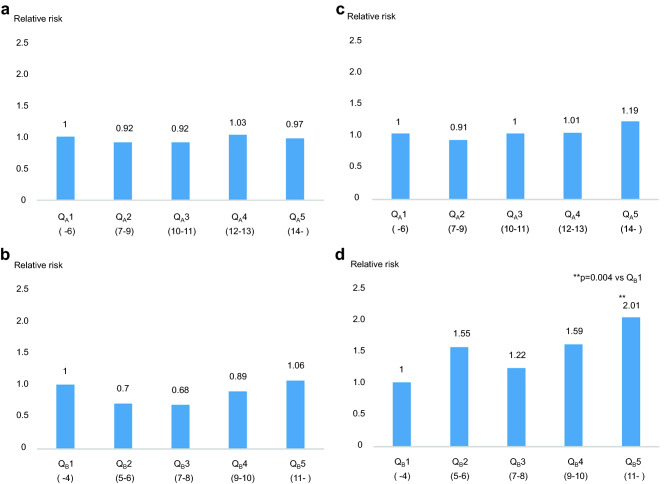
Relative risk of mortality. (**a**) The total number of prescribed medicines in the unadjusted analysis. (**b**) The number of prescribed non-essential medicines in the unadjusted analysis. (**c**) The total number of prescribed medicines in the adjusted analysis. (**d**) The number of prescribed non-essential medicines in the adjusted analysis. (**c**,**d**) were adjusted for age, sex, the duration of dialysis, dialysis time, diabetes mellitus, history of ischemic heart diseases, stroke history, cardiothoracic ratio, dry weight, systolic blood pressure before hemodialysis, hemoglobin, serum albumin, serum corrected calcium, phosphate, intact parathyroid hormone, blood urea nitrogen, serum creatinine, vitamin D use (irrespective of oral or intravenous), and phosphate binders. (**a**–**c**) There was no significant difference among the groups divided by quintiles. (**d**) There was a significant difference between the lowest quintile and the highest quintile (p = 0.004), and the relative risk of the patients in the highest quintile was two times higher than that of patients in the lowest quintile. Statistical analyses were conducted with Cox proportional hazard models using the JMP Pro 15.0.0 (3903308).

Next, we categorized the patients according to the quintile of the total number of prescribed medicines (Q_A_1–Q_A_5) and prescribed non-essential medicines (Q_B_1–Q_B_5), with Q_A_1 and Q_B_1 set as references. In the unadjusted analysis, there was no significant difference in the risk of mortality among Q_A_1–Q_A_5 and among Q_B_1–Q_B_5 (Fig. 3a-b). In the adjusted analysis (for the same factors as those in the multivariable Cox regression analyses), there was no significant difference among patients in Q_A_1–Q_A_5 (Fig. 3c). Although there was no significant difference in the risk of mortality between Q_B_1 and Q_B_2 (p = 0.08), Q_B_3 (p = 0.42), and Q_B_4 (p = 0.07), the adjusted risk was significantly different between patients in Q_B_1 and those in Q_B_5 (p = 0.004). In addition, the risk of mortality was twofold higher in patients in Q_B_5 than in those in Q_B_1 (Fig. 3d).

## Discussion

This retrospective cohort study elucidated the association between polypharmacy and prognosis in patients on HD and found that although there was no significant association between the total number of prescribed medicines and prognosis, the number of prescribed non-essential medicines was associated with prognosis after adjusting for clinically important confounding factors.

Older adults are particularly vulnerable to drug-drug interactions because they often have multiple chronic medical conditions that require multiple drug therapies. Globally, patients on HD are generally of advanced age^18^, which underlines the importance of polypharmacy as a crucial practice among patients on HD. Additionally, there is an increased tendency for prescribing new medicines to the patients on HD. For example, calcimimetics^19^, newly emerged phosphate binders^20^, and hypoxia-inducible factor prolyl hydroxylase enzyme inhibitors^21^ have been used in patients on HD in the last 10 years. Furthermore, new-generation antidiabetics, such as dipeptidyl peptidase-4 inhibitors^14^, can be prescribed safely in patients on maintenance dialysis. In this study, ischemic heart disease and diabetes were associated with taking ≥ 10 medicines.

The risk of drug-drug interactions is substantially increased when multiple drugs are administered^22^. Drug interactions can be induced by several mechanisms such as inhibition or induction of drug transporters, chelation, adsorption, and protein binding^23^. Furthermore, cytochrome P450 enzymes are associated with drug-drug interactions. Among more than 50 P450 enzymes, six enzymes (for example CYP3A4 and CYP2D6) are responsible for metabolizing 90% of drugs, which can competitively inhibit drug metabolisms^24^. Moreover, health care professionals avoid prescribing drugs eliminated by kidney in patients on HD^25^. Therefore, drug-drug interactions may have a profound effect in patients on HD, and this should not be overlooked.

Multivariable Cox regression analyses in this study showed that antihypertensive medicines (such as ARBs and CCBs) improved the prognosis of patients on HD. Blood pressure management in patients on HD requires not only antihypertensive medicines but also other strategies, such as diet therapy, exercise, and adjusting dry weight^26,27^. However, hypertension guidelines recommend the concomitant use of antihypertensive agents when the target blood pressure is not achieved^28,29^. Additionally, antihypertensive drugs may have other favorable functions beyond lowering blood pressure, such as protecting the cardiovascular system^12,30,31^. Despite the lack of robust evidence, antidiabetic drugs and statins are expected to suppress cardiovascular complications in patients on HD. Our findings support that some medicines need to be continued irrespective of the total number of prescribed drugs to achieve good blood pressure, glycemic, and cholesterol control.

Some gastrointestinal medicines were associated with a poor prognosis in this study. More than 60% of patients were prescribed proton pump inhibitors (PPIs) or histamine 2 blockers, and nearly 50% of the patients were prescribed other gastrointestinal medicines, such as metoclopramide and mucosal protectants. In general, PPIs accelerate osteoporosis, which worsens prognosis in patients on HD^32^, particularly older patients^33^. In addition, PPIs increase the risk of cardiovascular disease^34^. Although PPIs protect against gastric ulcers in patients prescribed aspirin, a previous report showed that more than 25% of patients visiting a single HD center took PPIs for an unclear/unknown indication^2^. Some patients in the present study were prescribed metoclopramide regularly, which could increase the risk of drug-induced parkinsonism^35^. Although there was no significant association between sleeping medicines and prognosis in this study, a previous report showed that benzodiazepines affected prognosis in patients on HD^35^. Collectively, these findings support that it would be beneficial to review the significance of non-essential medicines before they are prescribed routinely.

Issues related to polypharmacy can be addressed through several strategies. First, evidence on the benefit of medicines in patients on HD should be considered. As clinical trials tend to exclude patients on HD, the results may not be applicable to these patients. For example, increasing evidence shows that warfarin for atrial fibrillation increases the risk of stroke among patients undergoing HD^37^. A previous study established an effective algorithm to reduce the number of prescribed medicines considering the evidence in patients on HD^2^. Second, it would be better to seek alternative methods to medications. For instance, lifestyle changes may allow patients to discontinue sleeping medicines and decrease intra-dialysis weight gain, which in turn will allow discontinuation of oral vasopressors. Finally, prioritizing prescription medicines is crucial. Physicians and pharmacists should re-evaluate the prescribed medicines considering the patient characteristics and present status, including patient satisfaction. For example, although oral vasopressors negatively correlated with prognosis in this study, even after adjustment for patient characteristics, these drugs are necessary for safe HD. The outcomes of our sensitivity analyses show limited significance of decreasing the number of prescribed medicines in patients with diabetes mellitus. On the other hand, it is advisable to consider decreasing the number of prescribed medicines in patients with ischemic heart diseases. In this cohort, patients with ischemic heart diseases tended to be on dual antiplatelet therapy (DAPT). Although DAPT is mandatory for several months in patients with implanted drug-eluting stents, long-term antiplatelet therapy increases the risk of hemorrhagic complications, particularly in patients on HD^38,39^.

Several steps need to be implemented to address issues related to polypharmacy in patients on HD in the future. Although an effective prescription algorithm for patients on HD has been proposed^2^, its efficacy needs to be validated in large-scale studies. Additionally, it may be possible to study drug-drug interactions that consider individual patient characteristics, and use artificial intelligence to design appropriate formulations^40^. The increasing cost of medicine expenditure highlights the importance of decreasing the use of unnecessary drugs, and some limitations need to be proposed to curb the medical cost in HD^18^.

This study has some limitations. First, we only investigated the prescription profiles at the beginning of this study; the prescribed medicines could not be followed up during the observation period due to patients’ death. This could have led to a different interpretation of the results. Second, we could not assess drug-drug interactions using the multivariable regression models. We only adjusted for vitamin D and phosphate binders as they are most commonly used in HD. Third, the number of available medicines increased after the study period. For example, the high proportion of patients prescribed calcimimetics could be due to the recent availability of newer calcimimetics, such as evocalcet. Thus, the number of prescribed medicines might have increased during the observation period. Finally, we only focused on oral drugs and did not include intravenous medicines because some erythropoietin agents are only used once a month and are not administered daily. Almost all the drugs were used during HD; hence we excluded intravenous medicines to simplify the analysis. Moreover, we did not consider the dose of the medicines, which can affect the results. The association between polypharmacy and the dose of medicines and pill burdens should be evaluated in the future.

## Conclusion

Although the total number of prescribed oral medicines does not correlate with prognosis in patients on maintenance HD, the number of prescribed non-essential medicines impacts prognosis. This is because patients on maintenance HD have clinical presentation of highest complexity compared to patients without renal failure, and it is difficult to simplify the association between polypharmacy and patient prognosis in patients on HD. Consequently, medicines should be prescribed with caution in patients on HD, the prescriptions should be reviewed regularly, and the benefit should be balanced with the adverse effects.

## Methods

### Study design and patients

This retrospective study evaluated patients who underwent maintenance HD (3 sessions a week for > 3 months) at Nagasaki Renal Center between July 2011 and June 2012. The inclusion criterion was an age of at least 20 years. We excluded patients who did not undergo routine examinations in their birth month (e.g., blood tests) during the study period due to death or transfer to another facility. The patients were followed up until June 2021.

### Data collection

Data on baseline patient characteristics, such as age, sex, duration of dialysis, blood tests, pre-existing complications, and medications were obtained from electronic medical records in their birth months from July 2011 to June 2012. Only the number of oral medicines prescribed regularly was included in the analysis, and the doses and the number of pills were not considered. Over-the-counter drugs and prescribed medicines from other facilities were not counted. Antihypertensive drugs, diuretics, antidiabetics, and statins were categorized as essential medicines since these medicines reportedly exert favorable effect on the prognosis of patients on HD^11–16^. Moreover, our unpublished data showed that the number of antihypertensive medicines and diuretics positively correlated with patient prognosis in this cohort^17^. The total number of prescribed essential and non-essential oral medicines was used to elucidate the effect on prognosis.

### Statistical analyses

Categorical variables are represented as numbers (%), while continuous variables are presented as the mean ± standard deviation. Normally distributed data are displayed as the median (interquartile range). Continuous variables were compared using the *t*-test and Mann–Whitney U test, while categorical variables were evaluated using the chi-square test. The patients were divided into two groups based on the mean number of total prescribed medicines and non-essential medicines, and logistic regression analysis was performed to identify the patient characteristics that affected the number of prescribed medicines. Moreover, a survival analysis was performed between the two groups using the log-rank test. Additionally, another survival analysis between patients with and without CCBs was performed to show an example that essential medicines had a prognostic impact.

To calculate adjusted relative risks of the numbers of prescribed medicines, the patients were divided according to the quintile of the total number of prescribed medicines or the total number of prescribed non-essential medicines. Univariable and multivariable Cox proportional hazards analyses were also performed. To elucidate the effect of the number of prescribed medicines, Model 1 included the total number of prescribed medicines and was adjusted for clinically important factors, namely, age, sex, dialysis duration, dialysis time, diabetes mellitus, history of ischemic heart disease, stroke history, cardiothoracic ratio, dry weight, systolic blood pressure before HD, hemoglobin, serum albumin, serum corrected calcium, phosphate, intact parathyroid hormone, blood urea nitrogen, serum creatinine, vitamin D use (irrespective of oral or intravenous), and phosphate binders. Model 2 was used to elucidate the association between the number of prescribed non-essential medicines and prognosis adjusted for the same parameters. For sensitivity analyses, we assigned patients into two groups based on the presence of diabetes mellitus or ischemic heart disease. Multivariable Cox proportional hazards analyses included the age, sex, dialysis duration, dialysis time, cardiothoracic ratio, serum albumin, serum phosphate, serum creatinine, and the number of non-essential medicines.

The association between each medicine category and prognosis was evaluated by adjusting the parameters described above. Missing data were removed from the analyses. All statistical analyses were performed using the JMP Pro 15.0.0 (3903308) (SAS Institute Inc., Cary, NC, USA. https://www.jmp.com/en_my/software/new-release/new-in-jmp-and-jmp-pro.html). P < 0.05 was considered statistically significant.

### Ethics

This study was approved by the Clinical Research Ethics Committee of Nagasaki Renal Center (Nagasaki, Japan) (21010) and was conducted in accordance with the 1964 Declaration of Helsinki and its subsequent amendments. The need for informed consent was waived by Clinical Research Ethics Committee of Nagasaki Renal Center (Nagasaki, Japan) owing to the retrospective study design and use of anonymized data.

## Supplementary Information

Supplementary Information.

## Data Availability

The datasets analyzed during the current study are available from the corresponding author upon reasonable request.

## References

[1] Battistella M, Ng P (2020). Addressing polypharmacy in outpatient dialysis units. Clin. J. Am. Soc. Nephrol..

[2] McIntyre C, McQuillan R, Bell C, Battistella M (2017). Targeted deprescribing in an outpatient hemodialysis unit: A quality improvement study to decrease polypharmacy. Am. J. Kidney Dis..

[3] Oosten, M. J. M*. et al.* Polypharmacy and medication use in patients with chronic kidney disease with and without kidney replacement therapy compared to matched controls. *Clin. Kidney J.* 1–36 (2021). 10.1093/ckj/sfab120 PMC8690067 34950462

[4] St Peter WL (2015). Management of polypharmacy in dialysis patients. Semin. Dial..

[5] Roux-Marson C (2020). Medication burden and inappropriate prescription risk among elderly with advanced chronic kidney disease. BMC Geriatr..

[6] Tonelli M (2018). Comparison of the complexity of patients seen by different medical subspecialists in a universal health care system. JAMA Netw. Open..

[7] Nitta K (2020). Annual dialysis data report for 2018, JSDT Renal Data Registry: Survey methods, facility data, incidence, prevalence, and mortality. Ren. Replace. Ther..

[8] Masnoon N, Shakib S, Kalisch-Ellett L, Caughey GE (2017). What is polypharmacy? A systematic review of definitions. BMC Geriatr..

[9] Leelakanok N, Holcombe AL, Lund BC, Gu X, Schweizer ML (2017). Association between polypharmacy and death: A systematic review and meta-analysis. J. Am. Pharm. Assoc..

[10] Toida, T. *et al.* Impact of polypharmacy on all-cause mortality and hospitalization in incident hemodialysis patients: A cohort study. *Clin. Exp. Nephrol.* (2021). 10.1007/s10157-021-02094-9 34129133

[11] Inrig JK (2010). Antihypertensive agents in hemodialysis patients: A current perspective. Semin. Dial..

[12] Tepel M, Hopfenmueller W, Scholze A, Maier A, Zidek W (2008). Effect of amlodipine on cardiovascular events in hypertensive haemodialysis patients. Nephrol. Dial. Transplant..

[13] Kalantar-Zadeh K (2007). A1C and survival in maintenance hemodialysis patients. Diabetes Care.

[14] Ito M (2011). The dipeptidyl peptidase-4 (DPP-4) inhibitor vildagliptin improves glycemic control in type 2 diabetic patients undergoing hemodialysis. Endocr. J..

[15] Hsiao PJ (2017). Impact of the use of anti-diabetic drugs on survival of diabetic dialysis patients: A 5-year retrospective cohort study in Taiwan. Clin. Exp. Nephrol..

[16] Krane V (2016). Long-term effects following 4 years of randomized treatment with atorvastatin in patients with type 2 diabetes mellitus on hemodialysis. Kidney Int..

[17] Ota Y (2019). Effect of statin on life prognosis in Japanese patients undergoing hemodialysis. PLoS ONE.

[18] Himmelfarb J, Vanholder R, Mehrotra R, Tonelli M (2020). The current and future landscape of dialysis. Nat. Rev. Nephrol..

[19] Pereira L, Meng C, Marques D, Frazão JM (2018). Old and new calcimimetics for treatment of secondary hyperparathyroidism: Impact on biochemical and relevant clinical outcomes. Clin. Kidney J..

[20] Cozzolino M, Galassi A, Ciceri P (2021). Do we need new phosphate binders in dialysis?. Clin. Kidney J..

[21] Gupta N, Wish JB (2017). Hypoxia-inducible factor prolyl hydroxylase inhibitors: A potential new treatment for anemia in patients With CKD. Am. J. Kidney Dis..

[22] Al-Ramahi R (2016). Evaluation of potential drug- drug interactions among Palestinian hemodialysis patients. BMC Nephrol..

[23] Jia J (2009). Mechanisms of drug combinations: Interaction and network perspectives. Nat. Rev. Drug Discov..

[24] Lynch T, Price A (2007). The effect of cytochrome P450 metabolism on drug response, interactions, and adverse effects. Am. Fam. Phys..

[25] Whittaker CF, Miklich MA, Patel RS, Fink JC (2018). Medication safety principles and practice in CKD. Clin. J. Am. Soc. Nephrol..

[26] Agarwal R (2014). Assessment and management of hypertension in patients on dialysis. J. Am. Soc. Nephrol..

[27] Taniyama Y (2016). Management of hypertension for patients undergoing dialysis therapy. Ren. Replace. Ther..

[28] Unger T (2020). 2020 International Society of Hypertension Global Hypertension Practice Guidelines. Hypertension.

[29] Kario K (2019). Key points of the 2019 Japanese Society of Hypertension guidelines for the management of hypertension. Korean Circ. J..

[30] Sarafidis PA (2017). Hypertension in dialysis patients: A consensus document by the European Renal and cardiovascular Medicine (EURECA-m) working group of the European Renal Association-European Dialysis and Transplant Association (ERA-EDTA) and the hypertension and the Kidney working group of the European Society of Hypertension (ESH). Nephrol. Dial. Transplant..

[31] Takahashi A (2006). Candesartan, an angiotensin II type-1 receptor blocker, reduces cardiovascular events in patients on chronic haemodialysis—A randomized study. Nephrol. Dial. Transplant..

[32] Lazarus B, Grams ME (2018). Proton pump inhibitors in kidney disease. Clin. J. Am. Soc. Nephrol..

[33] Mittalhenkle A, Gillen DL, Stehman-Breen CO (2004). Increased risk of mortality associated with hip fracture in the dialysis population. Am. J. Kidney Dis..

[34] de Francisco ALM (2018). Proton pump inhibitor usage and the risk of mortality in hemodialysis patients. Kidney Int. Rep..

[35] Wang IK (2014). Increased risk of Parkinson’s disease in patients with end-stage renal disease: A retrospective cohort study. Neuroepidemiology.

[36] Winkelmayer WC, Mehta J, Wang PS (2007). Benzodiazepine use and mortality of incident dialysis patients in the United States. Kidney Int..

[37] Chan KE, Lazarus JM, Thadhani R, Hakim RM (2009). Warfarin use associates with increased risk for stroke in hemodialysis patients with atrial fibrillation. J. Am. Soc. Nephrol..

[38] Chen YT (2017). Dual antiplatelet therapy and clinical outcomes after coronary drug-eluting stent implantation in patients on hemodialysis. Clin. J. Am. Soc. Nephrol..

[39] Yamashita A (2021). Correlation between a bedridden status and the long-term outcome in hemodialysis patients after intracerebral hemorrhaging. Intern. Med..

[40] Ryu JY, Kim HU, Lee SY (2018). Deep learning improves prediction of drug–drug and drug–food interactions. Proc. Natl. Acad. Sci. U. S. A..

