# Supplementary material for: Prognostic impact of polypharmacy by drug essentiality in patients on hemodialysis
Source: Sci Rep. 2021 Dec 20;11:24238. doi: 10.1038/s41598-021-03772-0 (PMC8688458; doi:10.1038/s41598-021-03772-0)
Supplement: Supplementary file 1 — Supplementary Information. [file 41598_2021_3772_MOESM1_ESM.docx]

**Supplementary Table 1. Patient characteristics by the number of prescribed essential medicines**

|  | Less than 8  (N=163) | At least 8  (N=176) | p-value |
| --- | --- | --- | --- |
| Age (years) | 67.1±13.9 | 67.6±12.8 | 0.70 |
| Male (%) | 57.7 | 56.8 | 0.87 |
| Dialysis vintage^a^ (years) | 3.5 (1.3–8.6) | 5.8 (2.4–12.6) | <0.001 |
| Dialysis time^a^ (h) | 4 (3–4) | 4 (3–4) | 0.63 |
| Hypertension (%) | 84.7 | 84.6 | 0.98 |
| Diabetes mellitus (%) | 31.3 | 37.5 | 0.23 |
| Ischemic heart disease (%) | 27.0 | 40.9 | 0.007 |
| Valve replacement therapy (%) | 2.5 | 4.6 | 0.29 |
| Cerebral hemorrhage (%) | 8.6 | 4.6 | 0.13 |
| Cerebral infarction (%) | 25.8 | 23.9 | 0.69 |
| Arteriosclerosis obliterans (%) | 13.5 | 19.9 | 0.11 |
| Cardiothoracic ratio (%) | 51.6±6.1 | 52.7±5.4 | 0.07 |
| Dry weight (kg) | 51.6±10.6 | 52.5±11.4 | 0.43 |
| Systolic blood pressure (mmHg) | 149±24 | 150±25 | 0.74 |
| Left ventricular ejection fraction (%) | 65±10 | 65±10 | 0.83 |
| Hemoglobin (g/dL) | 10.7±1.3 | 10.9±1.4 | 0.13 |
| Ferritin^a^ (ng/mL) | 66.9 (25.9–166.5) | 63.3 (22.7–201.1) | 0.84 |
| Transferrin saturation (%) | 26.2±15.0 | 23.7±12.9 | 0.11 |
| Albumin (g/dL) | 3.5±0.4 | 3.6±0.4 | 0.17 |
| Corrected calcium (mg/dL) | 9.3±0.8 | 9.2±0.8 | 0.73 |
| Phosphate (mg/dL) | 5.5±1.7 | 5.7±1.6 | 0.28 |
| Intact-parathyroid hormone (pg/mL) | 72 (28–153) | 76 (28–154) | 0.93 |
| Alkaline phosphatase (IU/L) | 237 (191–324) | 259 (192–345) | 0.27 |
| Blood urea nitrogen (mg/dL) | 66.7±19.0 | 69.4±17.3 | 0.17 |
| Creatinine (mg/dL) | 9.8±3.5 | 10.7±3.3 | 0.02 |
| Total cholesterol (mg/dL) | 157±37 | 165±37 | 0.04 |
| Triglycerides (mg/dL) | 61 (82–129) | 98 (69–135) | 0.13 |
| C-reactive protein^a^ (mg/dL) | 0.19 (0.07–0.59) | 0.16 (0.07–0.46) | 0.73 |

Data are expressed as the mean ±standard deviation, ^a^ median (interquartile range)

The t-test or Mann-Whitney U test were used in the analysis.

**Supplementary Table 2. Trends in prescription by the number of prescribed medicines**

|  | Less than 10  (N=151) | At least 10  (N=188) | p-value |
| --- | --- | --- | --- |
| Erythropoiesis stimulating agents ^a^ (IU/week) | 4500 (2000–9000) | 4500 (2000–8000) | 0.73 |
| Iron including oral and i.v. (%) | 19.2 | 19.2 | 0.99 |
| Vitamin D including oral and i.v (%) | 60.7 | 71.9 | 0.03 |
| Angiotensin receptor blockers (%) | 33.8 | 47.3 | 0.01 |
| Beta blockers (%) | 14.6 | 23.4 | 0.04 |
| Calcium blockers (%) | 43.1 | 55.3 | 0.02 |
| Diuretics (%) | 25.8 | 41.0 | 0.003 |
| Other antihypertensive drugs | 4.0 | 20.7 | <0.001 |
| Anti-platelet drugs (%) | 20.5 | 51.6 | <0.001 |
| Warfarin (%) | 2.7 | 10.6 | 0.003 |
| Any phosphate binders (%) | 51.0 | 62.8 | 0.03 |
| Calcium carbonate (%) | 38.4 | 54.8 | 0.003 |
| Lanthanum carbonate (%) | 23.8 | 36.7 | 0.01 |
| Sevelamer (%) | 4.6 | 1.6 | 0.10 |
| Cinacalcet (%) | 14.6 | 17.6 | 0.46 |
| Oral antidiabetic drug (%) | 5.3 | 13.8 | 0.007 |
| Proton pump inhibitors | 30.5 | 46.8 | 0.002 |
| Histamine2 blockers | 21.9 | 26.6 | 0.31 |
| Other gastrointestinal medicines | 33.1 | 54.8 | <0.001 |
| Laxatives | 37.8 | 61.2 | <0.001 |
| Phosphate binders | 51.0 | 62.8 | 0.03 |
| Sleeping medicines | 17.9 | 29.8 | 0.01 |
| Anti-epileptic, parkinsonism, and depressant medicines | 9.3 | 24.5 | <0.001 |
| Statins (%) | 10.6 | 18.6 | 0.04 |
| Steroids (%) | 3.3 | 5.3 | 0.37 |
| Oral vasopressors | 20.5 | 31.4 | 0.02 |

Data are expressed as the mean ±standard deviation, ^a^ median (interquartile range)

The t-test or Mann-Whitney U test was used for the analysis.

The doses of darbepoetin alfa and epoetin beta are converted into epoetin doses at a ratio of 1:200.

IV: intravenous injection

**Supplementary Table 3. Prescription ratio by drug categories in the overall population**

|  | 1 | At least 2 | None |
| --- | --- | --- | --- |
| ARBs (%) | 41 | 0.6 | 59 |
| ACE inhibitors (%) | 1 | 0 | 99 |
| Beta blockers (%) | 19 | 0 | 81 |
| Calcium blockers (%) | 40 | 10 | 50 |
| Diuretics (%) | 30 | 4 | 66 |
| Other antihypertensive medicines (%) | 11 | 2 | 87 |
| Antiplatelets (%) | 29 | 9 | 62 |
| Warfarin (%) | 7 | 0 | 93 |
| Antidiabetics (%) | 8 | 2 | 90 |
| Proton pump inhibitors (%) | 40 | 0 | 60 |
| Histamine2 blockers (%) | 24 | 0 | 76 |
| Other gastrointestinal medicines (%) | 36 | 9 | 55 |
| Laxatives (%) | 29 | 21 | 49 |
| Phosphate binders (%) | 44 | 13 | 42 |
| Vitamin D (%) | 39 | 0 | 61 |
| Cinacalcet (%) | 16 | NA | 64 |
| Sleeping medicines (%) | 18 | 6 | 76 |
| Anti-epileptic, parkinsonism, and depressants medicines (%) | 14 | 4 | 82 |
| Anti-dementia medicines (%) | 4 | 0 | 96 |
| Oral iron medicines (%) | 2 | 0 | 98 |
| Statin (%) | 15 | 0 | 85 |
| Steroid (%) | 4 | 0 | 96 |
| Urologic agents (%) | 4 | 0.6 | 95 |
| Digitalis preparations (%) | 2 | 0 | 98 |
| Vasopressors (%) | 14 | 13 | 73 |

ARBs, angiotensin receptor blockers; ACE, angiotensin-converting enzyme; NA, not available

At least two means that patients took two and two and more kinds of medicines categorized the group. None means patients did not take the medicines categorized in the groups.

**Supplementary Table 4. Logistic regression model for patients prescribed at least 10 medicines**

|  | Univariable | | | Multivariable | | |
| --- | --- | --- | --- | --- | --- | --- |
|  | OR | 95% CI | p-value | OR | 95% CI | p-value |
| Age/year | 1.00 | 0.98–1.01 | 0.73 |  |  |  |
| Male vs female | 0.93 | 0.60–1.43 | 0.73 |  |  |  |
| Dialysis vintage/year | 1.02 | 0.99–1.04 | 0.26 |  |  |  |
| Dialysis time/h | 1.12 | 0.78–1.62 | 0.53 |  |  |  |
| Diabetes mellitus | 2.16 | 1.35–3.44 | 0.001 | 1.98 | 1.22–3.22 | 0.005 |
| Ischemic heart disease | 2.23 | 1.39–3.57 | <0.001 | 2.00 | 1.24–3.25 | 0.004 |
| Valve replacement | 1.63 | 0.48–5.53 | 0.42 |  |  |  |
| Cerebral hemorrhage | 0.65 | 0.27–1.55 | 0.33 |  |  |  |
| Cerebral infarction | 1.10 | 0.67–1.80 | 0.72 |  |  |  |
| Arteriosclerosis obliterans | 1.60 | 0.89–2.90 | 0.12 |  |  |  |
| Cardiothoracic ratio/% | 1.03 | 0.99–1.07 | 0.09 | 1.03 | 0.99–1.07 | 0.12 |
| Dry weight/kg | 1.01 | 0.99–1.03 | 0.29 |  |  |  |
| Systolic BP/10 mmHg | 1.09 | 1.00–1.19 | 0.06 | 1.06 | 0.97–1.17 | 0.21 |
| Left ventricular ejection fraction/% | 1.00 | 0.98–1.03 | 0.69 |  |  |  |

Significant parameters in the univariable analyses (i.e., those with a p-value <0.10) were included in the multivariable logistic regression model.

OR, odds ratio; CI, confident interval; BP, blood pressure

**Supplementary Table 5. Univariable Cox regression model for prognosis**

| Factor | Value | | |
| --- | --- | --- | --- |
|  | HR | 95% CI | p-value |
| Age/years | 1.07 | 1.05–1.08 | <0.001 |
| Male vs female | 1.10 | 0.84–1.42 | 0.50 |
| Dialysis vintage/year | 0.96 | 0.94–0.98 | <0.001 |
| Dialysis time/hour | 0.33 | 0.26–0.43 | <0.001 |
| Diabetes mellitus | 1.79 | 1.37–2.33 | <0.001 |
| Ischemic heart disease history | 1.20 | 0.92–1.58 | 0.18 |
| Stroke history | 1.81 | 1.38–2.34 | <0.001 |
| Cardiothoracic ratio/% | 1.09 | 1.06–1.11 | <0.001 |
| Dry weight/kg | 0.96 | 0.95–0.98 | <0.001 |
| Systolic blood pressure/10 mmHg | 0.98 | 0.93–1.04 | 0.51 |
| Hemoglobin/g/dL | 0.87 | 0.78–0.96 | <0.001 |
| Albumin/g/dL | 0.20 | 0.15–0.27 | <0.001 |
| Corrected calcium/mg/dL | 1.10 | 0.91–1.32 | 0.31 |
| Phosphate/mg/dL | 0.85 | 0.78–0.93 | <0.001 |
| Intact-parathyroid hormone/10 pg/mL | 0.97 | 0.96–0.99 | <0.001 |
| Blood urea nitrogen/10 mg/dL | 0.86 | 0.80–0.93 | <0.001 |
| Creatinine/mg/dL | 0.81 | 0.78–0.85 | <0.001 |
| Vitamin D | 0.64 | 0.49–0.84 | 0.002 |
| Phosphate binders | 0.37 | 0.29–0.49 | <0.001 |
| Total number of drugs | 0.99 | 0.96–1.02 | 0.56 |
| No. of non-essential drugs | 1.01 | 0.98–1.05 | 0.50 |

HR: hazard ratio; CI, confident interval

**Supplementary Table 6. Multivariable Cox proportional regression models on patients with or without diabetes**

|  | Patients with diabetes  (N=116) | | | Patients without diabetes  (N=223) | | |
| --- | --- | --- | --- | --- | --- | --- |
|  | HR | 95% CI | p-value | HR | 95% CI | p-value |
| Age/years | 1.03 | 1.01–1.05 | 0.01 | 1.05 | 1.03–1.07 | <0.001 |
| Male vs female | 1.68 | 1.00–2.81 | 0.05 | 1.52 | 1.03–2.24 | 0.03 |
| Dialysis vintage/year | 0.97 | 0.91–1.04 | 0.45 | 1.01 | 0.99–1.03 | 0.43 |
| Dialysis time/hour | 0.56 | 0.35–0.89 | 0.01 | 0.62 | 0.40–0.94 | 0.02 |
| Stroke history | 1.45 | 0.92–2.27 | 0.11 | 1.37 | 0.91–2.06 | 0.13 |
| Cardiothoracic ratio/% | 1.05 | 1.00–1.09 | 0.04 | 1.05 | 1.02–1.08 | 0.004 |
| Albumin/g/dL | 0.50 | 0.30–0.85 | 0.01 | 0.21 | 0.12–0.40 | <0.001 |
| Phosphate/mg/dL | 0.98 | 0.85–1.11 | 0.71 | 1.26 | 1.09–1.45 | <0.001 |
| Creatinine/mg/dL | 0.92 | 0.85–1.00 | 0.06 | 0.90 | 0.84–1.00 | 0.04 |
| No. of non-essential drugs. | 0.99 | 0.92–1.05 | 0.66 | 1.10 | 1.05–1.16 | <0.001 |

HR: hazard ratio; CI, confident interval

**Supplementary Table 7. Multivariable Cox proportional regression models on patients with or without ischemic heart diseases**

|  | Patients with ischemic heart diseases  (N=115) | | | Patients without ischemic heart diseases  (N=224) | | |
| --- | --- | --- | --- | --- | --- | --- |
|  | HR | 95% CI | p-value | HR | 95% CI | p-value |
| Age/years | 1.02 | 0.99–1.06 | 0.12 | 1.04 | 1.03–1.06 | <0.001 |
| Male vs female | 2.72 | 1.55–4.76 | <0.001 | 1.40 | 0.96–2.04 | 0.08 |
| Dialysis vintage/year | 1.01 | 0.98–1.04 | 0.45 | 1.00 | 0.96–1.03 | 0.84 |
| Dialysis time/hour | 0.60 | 0.36–0.98 | 0.04 | 0.56 | 0.37–0.84 | 0.005 |
| Stroke history | 0.96 | 0.56–1.63 | 0.87 | 1.91 | 1.32–2.77 | <0.001 |
| Cardiothoracic ratio/% | 1.04 | 1.00–1.09 | 0.06 | 1.04 | 1.01–1.07 | 0.01 |
| Albumin/g/dL | 0.24 | 0.12–0.50 | <0.001 | 0.42 | 0.26–0.67 | <0.001 |
| Phosphate/mg/dL | 1.01 | 0.86–1.16 | 0.87 | 1.22 | 1.07–1.39 | 0.002 |
| Creatinine/mg/dL | 0.84 | 0.75–0.93 | <0.001 | 0.93 | 0.86–1.00 | 0.04 |
| No. of non-essential drugs. | 1.09 | 1.01–1.17 | 0.03 | 1.06 | 1.01–1.11 | 0.02 |

HR: hazard ratio; CI, confident interval
